# Re-entering obesity prevention: a qualitative-empirical inquiry into the subjective aetiology of extreme obese adolescents

**DOI:** 10.1186/1471-2458-14-977

**Published:** 2014-09-20

**Authors:** Matthias Braun, Johanna Schell, Wolfgang Siegfried, Manfred J Müller, Jens Ried

**Affiliations:** Chair of Systematic Theology II (Ethics), Philosophical Faculty and Department of Theology, Friedrich-Alexander-University Erlangen-Nuremberg, Kochstraße 6, 91054 Erlangen, Germany; Obesity-Rehabilitation-Centre Insula, Bischofswiesen, Insulaweg 1, 83483 Bischofswiesen, Germany; Institute of Human Nutrition, Faculty of Agricultural and Nutritional Sciences, Christian-Albrechts-University Kiel, Christian-Albrechts-Platz 4, 24118 Kiel, Germany

**Keywords:** Obesity, Prevention, Subjective aetiology, Ethics, Governance, Life-style intervention

## Abstract

**Background:**

While numerous studies highlight the relevance of socio-cultural factors influencing incidence and prevalence of obesity, only a few address how obese people perceive causes and prevention of or intervention for obesity. This study contributes to a more thorough understanding of subjective aetiologies and framing themes for a mainly understudied but promising field. Thus it may serve for the development of effective public health strategies to combat obesity.

**Methods:**

Autobiographically based in-depth interviews were conducted with 20 patients (adolescents and young adults) institutionalised in the obesity rehabilitation centre INSULA in Bischofswiesen (Germany). The data were analysed with Atlas.ti with regard to two main perspectives: (1) How the interviewees perceive ‘their’ obesity from a subjective point of view and (2) which conclusions they draw from their own ‘story’ concerning prevention/intervention strategies.

**Results:**

The interviewees did not indicate a clear starting point for their overweight. Nevertheless, certain life-events (e.g. divorce or illness of parents) were identified as catalysing weight gain. As a consequence of coping with distress, body weight rises rapidly and not continuously. Obesity was generally framed as a problem primarily located within the family and not in the wider environment. Corresponding to this, the family was identified as the main and most important addressee of preventive measures. The interviewees highlighted the importance of personal responsibility as a prerequisite for self-determined action against obesity, but denied any link between responsibility and guilt.

**Conclusions:**

This study contributes substantially to a broader perspective on the prevention of obesity. First, more attention has to be paid to the interactions of medical aspects and the social dimension of obesity. Second, prevention efforts should be more aware of the relevance of subjective aetiology when it comes to the definition of reasonable and effective governance strategies in tackling obesity. Third, current assumptions concerning the importance of personal responsibility for obesity prevention might underestimate the relevance of self-determined action of the obese.

## Background

Obesity remains a substantial health problem. For one thing this is shown by the current data of prevalence, for example, in Germany [1], Sweden [2] and Europe [3]. In addition the multitude and the severity of the co-morbidities associated with obesity as well as the consequential costs predicted for health care systems [4] create a high pressure for action in politics and society [5]. Despite the constantly growing number of studies and research, neither a broadly applicable and at the same time effective strategy of prevention nor a respective theory option has yet been developed [6]. On the one hand this arises from a too narrow focus on behavioural prevention in research. This especially holds for approaches that solely address the self-responsibility of the individual obese person without systematically taking the different social and environmental causation factors into account. On the other hand the research of the last years has shown that obesity is a phenomenon of enormous complexity [6], which can neither be portrayed in one-dimensional models nor can it be researched, analysed and fought against by a restricted spectrum of methods. Since the last few years however, these confines have been cut back step by step. Research increasingly tries to capture the complexity of obesity as a medical and social phenomenon [7]. Moreover, efforts were made to genuinely integrate approaches of environmental prevention [8]. Since these changes are fundamental adjustments of prevention research, it has to be awaited in how far these changes also affect the development of effective strategies of prevention. If the intertwining of medical and social factors is eventually understood as a systematic necessity, this will lead to a high demand for realigned research. As a consequence, the social dimension of obesity will increasingly be brought into focus. Having stated this, two fundamental desiderata can be identified: First, nowadays there are numerous studies on the meaning of socio-cultural and socio-economic factors of influence on the incidence as well as the prevalence of obesity [9]. However, it remains open whether any adjustment or manipulation of those socio-cultural and socio-economic factors would make a difference to very obese individuals. In fact, their subjective perspectives about themselves could be a higher obstacle to prevent or lose body weight than the socio-cultural and socio-economic factors [10]. The subjective perceptions and evaluations of their own genealogy of obesity can – according to the hypothesis of the present article – be essential for the efficiency of respective means of intervention. When developing a systematic approach for fighting obesity, the first requirement is to include the perspective of those who are affected.

The second desideratum, which is closely linked to the first, affects the use of methods. If the aspect of subjective perceptions is taken seriously, it will have immediate consequences for the further use of methods: A systematic integration of the perspectives of the affected is only partly possible if the sole focus lies on a quantitative-empirical research perspective. Thus qualitative-empirical methods should be chosen more often as they already have been in various medical-scientific contexts. In the field of clinical or psychiatric care, for example, this approach has been used for some years now. The instruments of qualitative research aim especially at capturing a broad range of subjective attitudes or perceptions in different environmental contexts. This is how they open up an in-depth dimension that can hardly be depicted by the sole use of quantitative methods [11]. Therefore narrative elements, which are gathered and analysed with the help of different tools, are an undeniable advantage of qualitative studies [12]. Apart from their inherent value, i.e. knowledge won by qualitative methods, the respective qualitative studies may also complement quantitative methods. In fact, they can identify questions and areas that have to be newly or more intensely researched.

Health-related research in general and obesity research in particular are increasingly using qualitative methods. Examples include the development or refinement of quantitative instruments [13, 14] as well as studies that focus on the interaction of patient and physician [15, 16]. A focus of qualitative research in the field of obesity can be found in those studies that deal with stigmatising experiences in different settings [17, 18]. Furthermore, first qualitative research studies have been conducted to explore lay perspectives of obesity causation [19–21]. A first meta-synthesis (the equivalent of a meta-analysis of quantitative data) of qualitative studies in the field of obese children and adolescents has recently been published [22]. It pays attention to the setting ‘therapy’ by examining the interaction between doctors or medical staff with obese children and adolescents as well as with their parents. In contrast, qualitative studies that concentrate on the area of “prevention” are found less frequently.

Against this background, the present study starts with a threefold hypothesis: First, the subjective reconstruction of the development of the individual obesity aetiology is understood as an essential factor leading towards a more detailed understanding of different obesity formation pathways. Although it is not assumed that a more detailed understanding of the subjective aetiology does directly lead to an increasing amount of effective and efficient preventive strategies, it would certainly ease this very process. Second, possible explanations for the ineffectiveness of prevention strategies that have been developed so far have to be worked out. In this context, particular importance has to be paid to the alleged difference between professionally diagnosed aetiologies and the subjective aetiologies. Thirdly, this study assumes that a detailed comprehension of differences between subjective and professionally diagnosed aetiologies can be gained by means of qualitative-empirical methods.

## Methods

For the present study autobiographically based interviews [23–25] were conducted, analysed and evaluated. This type of interview was chosen to gain a more thorough understanding of the importance of subjective aetiology. Additionally, this method offers the opportunity to analyse interactions between patient perspectives and its professionally diagnosed perspective. Finally, possible consequences for obesity prevention strategies can be found. The qualitative interview study was conducted with patients of the obesity rehabilitation centre INSULA (Adipositas-Reha-Zentrums Insula) in Bischofswiesen (Germany). The inquiry was exclusively based on participants who were already at an intervention setting when the interview was conducted. This special setting was chosen to scrutinise the perceptions and especially the retro-perspective evaluation of obese people who already have a lot of experience with different types of intervention measures and programmes. Three different appointments were held to interview all of the adolescents and young adults (N = 20). The participation in this inquiry was voluntary and unpaid. The study was advertised throughout the hospital facility by putting up invitations to the study on notice boards. Additionally the research project was introduced to the patients in a special information event conducted by the researchers. With regard to possible mental and emotional efforts all participants were informed that they could stop the interview at any point. Fortunately, all interviews were completed without any participant making use of this option. The study was performed with the approval (55_14B) of the ethics committee of Friedrich-Alexander-University Erlangen-Nuremberg. The participants received written informed consent for the participation in the study. Furthermore, the study has been developed and conducted according to the RATS guidelines.

The average age of the participants was 18.8 years (range: 16–25 years), the participants were patients in the clinic for an average of 99 days (range: 1–360 days) at the time of the interviews and their average BMI was 42.33 kg/m^2^ (range: 30.0-97.2 kg/m^2^). All participants could be classified as extreme obese patients (as they were above 35.0 kg/m^2^ at least at some point in their life). All of them have experienced the ineffectiveness of different prevention approaches and therapeutic regimes. They invested considerable efforts in decreasing weight and thus offered a broad amount of experiences and knowledge in the field of self-perception of incidence as well as the prevalence of their obesity. The participants’ level of education was also surveyed and segmented according to ISCED (see Table 1).

**Table 1:** **Overview data of the study**

Participants		
Total		20
Male		12
Female		8
**Age**		
Range (years)		16-25
Mean average age (years)		18.8
**Weight data**		
Ø BMI (start)		47.11
Ø BMI (current)		42.32
Ø duration of intervention (days)		14.14
**Highest (current) education level in ISCED categories**		
0-1	first stage basic education	2
2	lower secondary or second stage of basic education	9
3	upper secondary education	9
4	post-secondary, non-tertiary education	0
5	tertiary education	0

The interviews were autobiographic-narrative and problem-centred oriented, and they were conducted according to a standardised framing. With regard to the concepts of ‘grounded theory’, this framing wasn’t used to set a fixed agenda of questions but rather as a rough topic structure in order to warrant the comparability within the study. Therefore the interviews were divided into three parts: The first and at the same time most extensive part of the interviews served as the reconstruction of the environmental experiences of the participants with their usually extreme form of obesity. Special attention was paid to the participants’ subjective linking between certain life-events or past experiences with phases of considerable increase in weight. Similarly, their experiences in fields of life as family, school, work place and health care system in relation to their obesity were considered.

In the subsequent part of the interview, individual aspects of the first section were taken up more thoroughly and possible areas in which stigmatisation could have been perceived were sampled. Stigma and discrimination toward obese persons are surmised to pose numerous consequences for their psychological and physical health and thus seem to interfere with effective obesity intervention efforts [26, 27]. In order to support the narration and to create stimuli, a German translation of the Weight Self-Stigma Scale [28], including twelve statements about the subjective perspective on obesity, was applied in the interviews. The participants were free to add narrative elements (e.g. a report on experiences and perceptions) with their reflection on the statements. In the concluding part, the participants were encouraged to reflect their narrated experience in respect to possible conceptions of effective prevention strategies.

All interviews were digitally recorded. For analysing purposes, the data were at first made anonymous and afterwards transcribed. For reasons of comprehensibility the original transcripts will be presented prior to their English translation in the following part. The evaluation of the interviews was conducted detached from the interviewers in several independent steps. In order to extrapolate the different elements of the subjective obesity aetiology in the imaginations and narrations of the participants, two steps were undertaken. First, the data were theoretically coded [29, 30] and second, two themes were highlighted: on the one hand, the participants’ associations between gaining weight and certain live-events and experiences, on the other hand, how participants interpreted their experiences. One of the leading questions of the latter theme was for example: In how far and at which points do the participants see their ‘story’ as controllable and changeable? Special emphasis was put on the storytelling aspect [31], i.e. reconstructing the aetiology from the perspective of the individual. The study understands the particular stories and narrative patterns as conceptual frames for processing and articulating the subjective aetiology of those affected. Thus it links interpretative research with grounded theory [32, 33].

## Results and discussion

### The subjective aetiology of obesity

The analysis of the data leads to three essential results regarding the first focus of evaluation: The evaluation shows that the participants (1) could not give a genealogical starting point for their obesity, (2) they each identified different life events [34] as catalysts for the weight increase and (3) they understood obesity as a family problem in a threefold sense.

#### No genealogical starting point

First, neither of the participants could clearly identify a genealogical starting point for their weight gain. Obesity was rather experienced as something that has always been present:

*“Pummelig war ich eigentlich schon mein Leben lang. Das (2) begann schon bei der Geburt, wo ich .. über 5000 Gramm war” (C90GM5, l.011).*

*“I’ve always been chubby, actually my whole life. That (2) began when I was born when I .. had over 5,000 grams”*

In some cases participants were able to provide more precise periods of time in which they gained weight. However this did not change their assessment that overweight could not be attributed to an initial life event:

*“Also, .. ich war mit drei schon übergewichtig, also irgendwie immer” (J56CL1, l.017)*

*“Well, .. I was already overweight at three, actually I have somehow always been”*

Even if certain temporal markers were added, most of the time this had no real implication. They were just part of the subjective story line but were not understood as a genuine part as such:

*“Also, ich war schon immer dick, also auch als Kind, als Fünf-, Sechsjähriger hat das angefangen laut meinen Eltern” (A12ZY1, l.139)*

*“Well, I have always been big, even as a child, as a five-, six-year-old it started according to my parents”*

This result can be found throughout all samples and was independent of age, gender or level of education.

#### Life events as catalysts

Though no initial meaning was attributed to the life events for the genesis of obesity, they seem to function as catalysts for the further increase of weight in a significant way. These life events were perceived as fateful on the one hand, but were at the same time determined as possible chances of intervention. At this point, it is crucial to make clear that the participants did not primarily understand such means of intervention as interventions concerning their weight. Instead they understood their eating and mobility behaviour as reactions and strategies of coping with the problem seen as primarily responsible. By the participants, interventions were only considered as having a chance to succeed if they primarily focus on the priority impairments and thus only indirectly address disposing with the eating and mobility disorder.

The analysis shows the following relationships between the different life events and the weight increase: First, the individual life events may accumulate and cannot always be clearly separated from one another. Second, many participants considered excessive eating as an act of defiance towards the family and simultaneously as a very important factor in the respective story lines. Various possible events, such as experiencing domestic violence, the separation of the parents or the death of a family member were considered as leading to these acts of defiance:

*“… Erwartungen, die ich halt so .. an ’n Vater gestellt hab, also von – Vater hatte – (2) kam nie rüber – also Desinteresse v o n Vater, Missachtung von der Mutter, .. zum Teil auch häusliche Gewalt von der Mutter. (O23DY9, l.120-124)] und [.. Und deshalb wusst ich halt nie, wie ich’s verarbeiten sollte, außer halt .. mit ’m Essen und (2) meistens mehr als (2) eigentlich der Magen haben wollte (O23DY9, l.126-127).*

*“… expectations, which I had, as I said, .. of my father, well had from – father – (2) never came across – well disinterest f r o m father, disregard from the mother, .. partly domestic violence from the mother, too.] and [.. and therefore I, as I said, never knew, how I should process it, but, as I said, .. with eating and (2) most of the times actually more as (2) the stomach wanted to have.”*

*“… wir hatt’n nu’ häusliche Gewalt zuhause und dann be – hat mein Vater mich auch ’n paar Mal geschlagen, eben Johanna Jäckl 22. September 2014 12:29: weil.. er irgendwie ganz entsetzt war, wieso ich denn auf einmal so dick bin, .. ähm, und mit meiner Mutter, ähm, die hatte irgendwie so ’ne Angst, dass mich irgendwann selbst aufgebe, und das war halt das Körpergewicht oder, äh, s – das Indiz ((verhalten auflachend)) dafür. (J56CL1, S.6, Z.151-155.)] und [dann jedes Mal bei Familienfeiern und so, dann wurden wir praktisch in jedem Satz beleidigt wegen unsr – unsres Gewichtes … (J56CL1, S.7, Z.157-158)”*

*“…we had domestic violence at home and then sta - my father also hit me a couple of times, just because .. he was somehow really appalled, because I was suddenly this big, .. um, and with my mother, um, she somehow had this fear, that I could give up myself eventually, and for this was, as I said, the body weight or, uh, s - the indication ((restrained laughing))] and [then each time at family celebrations and so on, we were being insulted practically with each sentence because of ou - our weight…]”*

Excessive eating was often a means for compensation for these life events which were perceived as severe and threatening. While this behaviour was sometimes understood as open protest, for example to call for attention to the particular problem, it served at the same time as an internal strategy of dealing with the problem. Such a separation, however, proves to be rather artificial concerning the progress of the whole storyline. In fact, there are often transitions in the temporal perception of the respective modes of behaviour:

*“Ähm und (2) dann, äh, is mein .. Vater auf Kur gegangen, .. im selben Jahr und is dann, äh, nach der Kur, .. ähm – .. hat uns dann verlassen, also er hat sich von meiner Mutter getrennt und .. is weggezogen und, .. ähm, ja dann .. war sehr viel Trauer in der Familie und, äh, .. ich, ja, ich konnte (?den dann) nich so ganz bewältigen zu dem Zeitpunkt und hab mich dann sehr zurückgezogen, .. hab dann auch angefangen, exzessiv am Computer zu spielen, und, .. ähm, zu dem Zeitpunkt hatt ich au’ nicht mehr sehr viel soziale Kontakte wegen .. Vorfällen davor, .. wo sich mein Freundeskreis, äh, sehr ausgedünnt hat.] und [Hm, .. also, ich glaube, wenn (2) – wenn mein Vater damals, ä h, sich nich entschieden hätte dazu, ähm, .. uns zu verlassen, .. denk ich, wär ich auf ’m guten Weg gewesen, .. hm, nicht so viel zuzunehmen, weil i – ich war damals, .. sag mal, relativ schlank .. füm–, äh, für meine Verhältnisse und, .. ähm, ich bin ’n halbes Jahr aus ’er Kur draußen gewesen, es war – es war alles relativ gut gelaufen und, .. ähm, .. ja, aber ich bin halt, wie gesagt, in’n sehr tiefes Loch gefallen, als er hier so weg war, und, äh, (2) ich denke, wär das nich passiert, dann wär ich jetzt, äh, nich hier, dann hätt ich jetz wahrscheinlich ’ne Ausbildung und .. würde wahrscheinlich selbstständig leben.” (F49RT2, l.015& l.077)*

*“Um and (2) then, uh, my .. father went to a health resort, .. the same year and then he, uh, after the cure, .. um - .. left us then, he split up with my mother and .. moved away and, .. um, yes then .. there was a lot of sorrow in the family and, um, .. I, well, I could not really cope with it at that time and then I withdrew back into myself, .. started excessively playing at the computer, and, .. um, at that time I didn’t have much social contacts because of .. incidents before, .. where my circle of friends, uh, thinned out quite a lot.] and [Hm, .. well, I believe, if (2) – if my father had, uh, decided differently then, um, .. about leaving us, .. I think I would have been on a good way, .. hm, not to gain that much, because I – I was then, let’s say, relatively lean .. bym-, uh, by my standards and, .. um, I had been out of cure for half a year, it went – it went all relatively well and, .. um, .. well, but I did fall, as I said, into a very deep hole when he was gone here, and, uh, (2) I think if that didn’t happen, then I would, uh, not be here now, then I would probably have an apprenticeship now and .. would probably live on my own.”*

In the preceding quote the participant’s explanations patterns can definitely be understood as protest or a strategy of coping with the described life event. Depending on this differentiation of protest or coping strategy, distinct types of intervention were considered adequate. Besides the act of defiance towards their families, another explanatory model for the excessive consumption of food was to regard it as a reaction to changes in the respective stage of life. Changes of the familiar surroundings due to moving or school transition as well as the start of puberty were mentioned most frequently. Whereas the excessive consumption of food was often associated with particular life events, the lack of activity was not. Instead the latter was attributed to continua of experience. Thus, especially the persistent stigmatising experiences – and thus the co-occurrence of labelling, stereotyping, separation, status loss, and discrimination [35] – within the different life events were the trigger to shift the participants’ focus of interest and life into domestic or also digital worlds. Both, the decreased perception of offers for activity or eventually the active avoidance of any physical activity, were also linked to this. After describing these shifts into inactivity all participants mentioned their attempts to find ways back into activity. Four participants told about specific efforts like joining sports clubs or fitness clubs. However, they stopped their efforts after their environment acted negatively to their endeavours. Comments that were made by the immediate, and again especially domestic, setting were deemed particularly relevant. Possible verbal or gestural expressions of agents of the broader social setting were experienced as less grave.

Taken as a whole, it can be stated that different life events were on the one hand identified as starting points for enormous increase in weight. On the other hand, attempts to either stop further weight gain or starting weight loss were only considered to be reasonable and expedient if the obesity was directly linked to the previously described life events. This aspect was also confirmed by the fact that the participation in different medical programmes for intervention at the very time of these critical life events did not achieve any or just a negligible short-term effect: obviously, they did not address the actual problem nor did they identify it.

#### Obesity as a threefold family challenge

Closely connected to this second point, there is a third aspect, namely the participants’ view of obesity being mainly a family issue. However, such a localisation is also ambivalent. First and corresponding to numerous existing studies on the connection to maternal and paternal weight [36, 37] and the importance of the family’s socio-economic status [38, 39], all participants told about an accumulated occurrence of overweight in the family circle. This involved the closest as well as the extended family circle.

*“Äh, schon, .. also, hm, .. meine Mutter ist übergewichtig, meine Schwester auch .. und mein Bruder auch, aber die beiden haben das jetz auch hingekriegt mit Abnehmen .. u n d mein Vater, der is eigentlich auch ’n bisschen moppelig.” (G19MV4, l.113)*

*“Uh, even, .. well, hm, .. my mother is overweight, my sister too .. and my brother too, but both of them have by now managed to lose weight .. a n d my father, he’s actually also slightly pudgy.”*

Second, the view of obesity being a family issue is supported by the fact that all participants pointed out that, from their perspective, the family was the only reasonable setting where a strategy of prevention has to begin. Medical interventions as well as behavioural-preventive measures at kindergarten or at school were less appropriate according to the subjective perspective of those affected. Although they were regarded as important and expedient as flanking measures, they would not address the actual challenge. However, even if intervention within the realms of the family was existent, by focusing (too) strongly on the respective intervention a certain factor of resignation became apparent with the participants:

*“das Gewicht und auch das Essverhalten war eigentlich dann wirklich das einzige regelmäßige Thema, über das man so gesprochen hat.” (J56CL1, l.053)*

*“the weight and also the eating behavior was actually really the only regular topic about which you talked.”*

Third, this resignation also had its effect on the evaluation of suggestions for interventions outside of the family. For example, by launching a school intervention concerning the consumption of high-calorie drinks, the subject matter would have been omnipresent not only at the participants’ homes but in their every-day environment which thus constantly pointed at their “problem”.

The focus on the family as the primary setting of intervention, however, didn’t reveal anything about the affective or emotional proximity of the participants towards their families. It can rather be regarded as some relatively pragmatic information for the localisation of the source of obesity: Obesity as a domestic outgrowth. This means that the essential starting point for intervention has to be set within the family.

### Subjective aetiology and personal responsibility

Concerning the second evaluation focus, i.e. in how far the participants regarded their own story as changeable and shapeable, there are two substantial results. Firstly, overweight was understood as an essential marker of identity which they, if possible, didn’t want to lose completely.

*“es auch so ein bisschen, äh, das gehört auch zu mir, dass ich dick bin, also, ich in– identifizier mich wirklich damit, ähm, ich kann mir das jetz nich vorstellen, .. eben weil’s auch noch nie war, dünn zu sein, und ich mag es auch nich, meine Knochen zu spür’n und, ähm, .. genau deswegen würd ich eigentlich doch schon eher dazu tendieren, nich unter 90 zu kommen.” (J56CL1, l.184)*

*“it’s also a bit like, uh, it’s part of me that I’m big, well, I in- identify myself with that, um, I can’t imagine, .. precisely because it’s never been like that, like being thin, and I also don’t like it to feel my bones and, um, .. that’s exactly why I’d actually rather tend to not come below 90.”*

Despite of the existing awareness of their obesity, their own weight is understood as something that is part of their own identity. It presents a positive as well as a negative distinction from the physical as well as aesthetical norm which is characterised as the societal ideal. The participants – especially in the self-reflective parts of the inquiry – questioned vehemently which instance is actually authorised to determine what is to be regarded as still normal and what as already anomalous. Dealing with such social norms and images took up an essential part when forming their own identity. By negating the social weight norm, the affected marked a distinction that has been shown to be a constitutive element of the participants’ own social localisation.

Simultaneously, all participants insisted on their responsibility for their own health. Personal responsibility was viewed as an essential requirement for being able to intervene at all. The research furthermore showed the striking fact that according to the participants’ perspective the acceptance of responsibility for their own health is not connoted with the assumption of guilt.

*“Ja, ich hab mein Gewichtsproblem schon selbst verursacht, weil ich halt schon viel gegessen hab. (2) Ich fühl mich aber nich schuldig, .. weil .. das schadet ja eigentlich nur mir .. und .. ja, .. es is halt mein Problem.” (E15NU7, l.207)*

*“Yes, I’ve caused my weight problem myself because I’ve eaten quite a lot. (2) But I don’t feel guilty .. because .. that only Johanna Jäckl 22. September 2014 12:48: harms me.. and .. well, .. it’s just my problem.”*

*“Alle anderen hat’s eigentlich nich zu interessieren, weil das nich ihr Körper is, und mir selber – .. nein ((lacht verhalten auf)).” (A12ZY1, l.471)*

*“It’s none of anyone else’s business because it isn’t their body, and for myself Johanna Jäckl 22. September 2014 12:49: – ..no ((laughs in a noncommittal way)).”*

As clearly as the participants objected a socially ascribed guilt, they vehemently held onto their own responsibility for their overweight. Linking this aspect to the results above seems to put out an ambivalent and perhaps contradictory result. On the one hand, the participants viewed the genesis of their obesity as intrinsic to them as well as to extrinsic factors as different life events. At a first glance, it seems to fit in with such an interpretation that the role of the “scapegoat” is transferred to life events described above. However, this does not entail that the origin of the participants’ overweight fully falls outside their own area of responsibility.

*“.. das seh ich definitiv so, also, .. wer war’s denn sonst, ((verhalten auflachend)) also ich mein, .. hat mich ja niemand zum Essen gezwungen. ((stöhnt leicht auf))” (F49RT2, l.089)*

*“.. I definitely see it like that, well, .. who else was it, ((laughs in a noncommittal way)) well, I mean .. no one forced me to eat. ((moans quietly))”*

Undoubtedly there are supposed intrinsic or extrinsic factors which are partly responsible for the current obese situation of the participants. However, the participants insisted that the only way to deal with these “factors” was to refigure the modes in which they regard and handle them.

An additional factor with regard to self-perceived responsibility is that of the overall assumption that the overweight will not remain a life-long problem: rather it is seen a challenge which can be dealt with and for which the participants will assume responsibility.

### Résumé

The following results have to be pointed out as especially significant: Firstly, according to the perspective of those affected, overweight is regarded as something that has always been present and which has no clearly definable starting point. Secondly, different life events can however be understood as catalysts for the enormous gain of weight. Therefore it seems not to be appropriate to assume that there is a rectilinearly continuum from normal weight to extreme obesity. Rather the different life events seem to function as initiations for periods of accelerated weight gain. Thirdly, the participants consider obesity to be a domestic issue, both concerning the incidence and the possibilities of intervention. Fourthly, the results of the study show that the challenge in dealing with the own obesity can neither solely be solved by medical nor by social parameters, but is always entailed with the question about the participants’ self-conception and the norms they accept as valid. The fifth essential point regards the relationship of personal responsibility and guilt. In the context of this inquiry, no connection between taking up personal responsibility and assigning guilt emerged. On the contrary, the participants understood personal responsibility as an essential room for possibilities of the self-determined shaping of their own way of life. The results of the inquiry suggest the assumption that the participants consider themselves responsible for the increase of their weight without associating an accusation of guilt with it.

## Conclusions

Even though the setting of the study was in so far special as all participants were already at a point of institutionalised intervention when the inquiry was conducted, the results of this study can provide important additions and adjustments to the previous strategies in the field of obesity prevention.

Generally, the necessity for developing a systematic approach including both medical and social parameters for obesity prevention can be supported against the background of these results [40, 41]. This implies the importance to pay attention to the interaction between the medical and social dimensions of obesity but also to consider the social dimension (e.g. intra- and inter- familiar aspects, temporal shifts of self-explanation, different environmental contexts) itself. If the results of this study can further be verified, the unconscious refusal of not paying enough attention to this very interaction could play an important role in the explanation of the previous ineffectiveness of obesity prevention. This becomes especially apparent at the point when the study’s participants vehemently pointed out the domestic dimension of obesity. Concluding from this the following fields of concrete challenges can be identified:

1. The previous approaches of obesity prevention cannot be accused of not having recognised the importance of obesity as a domestic phenomenon, but most intervention studies, with their limited effectiveness, chose their starting point majorly in the obesogenic environment, and not in the families themselves. Of course, there are a few studies within the field of family prevention strategies [42] struggling with the possible effectiveness of such intervention programmes. But nevertheless these endeavours are primarily focusing on the medical aspects. Since those affected see obesity as a primarily domestic and not exclusively medical problem, this could be one explanation for the mostly ineffective methods of previous, especially extensive and unspecific intervention strategies. In addition this point is getting much more ambivalent when taking into account that the participants identified their families as their ‘obesogenic environment’. At the same time, they emphasize that it would make their situation even worse and complicated if obesity was directly tackled by different prevention or intervention measures. Therefore, a family prevention approach seems to be challenged to tackle obesity indirectly by focusing on the primary social – and intra-domestic – problems.
2. Floodlighting the previous aspect, the results draw attention to the fact that obesity itself is not the actual issue but rather particular life events. Problems concerning the development of the body weight happen to appear as accompanying and consecutive phenomena of life issues. If this observation holds true, this will have enormous consequences for the further research in the field of obesity prevention: the effectiveness of the preventive measures, for example, seems to be – largely – dependent on how far the respective recipients of the prevention see themselves as recipients of these interventions.
3. These considerations are closely related to the question about the objective of obesity prevention. If the first results of this study can be confirmed, the question concerning the norms governing the interventions will have to be asked all over again. Not only conceptual questions regarding the respective understanding of disease and health will have to be brought into focus [43], but also questions concerning the determination of the threshold level for necessary intervention. Focusing on factual medical parameters like the BMI or the relation of muscles towards fat mass means taking a high risk: it may lead to a paternalistic determination of interventional necessities without considering the particular life situation or the individual and perhaps deviant perceptions of a healthy body.
4. Throughout the research literature a connection between the reference to individual responsibility and a simultaneous attribution of guilt is pointed out consistently. However, this assumption can no longer be sustained in the context of this study’s results. One of the ambivalently discussed concepts in the current debate is the expression of personal responsibility since it is connected to the fear of an illegitimate attribution of guilt [44] concerning the individual. Also, it is associated with an increase in stigmatization, either of the implicit type – i.e. subconscious, automatic, and intuitive – or the explicit type – i.e. conscious, controllable, and reflective [45, 46]. The results of this study allow the conclusion that personal responsibility can be very well understood as a central concept for dealing with one’s own health. This is due to the fact that only by acknowledging responsibility, a self-determined individual that regards him- or herself responsible for his or her actions and behaviour can be acclaimed. Whereas in recent years there has been a shift in perspectives from a behavioural to a relational prevention in obesity prevention, it can now be questioned whether this shift was necessary: the phenomenon of obesity has proven to be even more complex and thus behavioural and relational prevention have had to be re-connected. In future, behavioural and relational prevention could merge in a way that enables us to create fields of action based on an individual determination of ‘risky’ life events and intervention strategies.
5. For further adjusting current as well as future prevention strategies, it would be worthwhile to widen the scope of research by including the perspective of those affected. Special attention should be paid to systematic consequences for obesity research. Nevertheless, these results also stress the requirement of an initial debate on both, the understanding as well as the scope of family prevention.
